# Caspase-3-independent apoptotic pathways contribute to interleukin-32γ-mediated control of *Mycobacterium tuberculosis* infection in THP-1 cells

**DOI:** 10.1186/s12866-015-0366-z

**Published:** 2015-02-21

**Authors:** Xiyuan Bai, William H Kinney, Wen-Lin Su, An Bai, Alida R Ovrutsky, Jennifer R Honda, Mihai G Netea, Marcela Henao-Tamayo, Diane J Ordway, Charles A Dinarello, Edward D Chan

**Affiliations:** Department of Medicine, Denver Veterans Affairs Medical Center, Denver, CO USA; Departments of Medicine and Academic Affairs, National Jewish Health, D509, Neustadt Building, 1400 Jackson Street, Denver, CO 80206 USA; Department of Medicine, University of Colorado School of Medicine, Aurora, CO USA; Division of Infectious Diseases, University of Colorado Denver Anschutz Medical Campus, Aurora, CO USA; Division of Pulmonary and Critical Care Medicine, Taipei Tzu Chi Hospital, Buddhist Tzu Chi Medical Foundation, New Taipei andTri-Service General Hospital; National Defense Medical Center, Taipei, Taiwan; Department of Internal Medicine and Radboud Center for Infectious Diseases, Radboud University Nijmegen Medical Center, Nijmegen, The Netherlands; Mycobacteria Research Laboratories, Department of Microbiology, Immunology and Pathology, Colorado State University, Fort Collins, CO USA

**Keywords:** Mycobacterium tuberculosis, Interleukin-32, Apoptosis, Programmed cell death, Caspase-1, Cathepsins, Apoptosis-inducing factor

## Abstract

**Background:**

Macrophages are the primary effector cells responsible for killing *Mycobacterium tuberculosis* (*MTB*) through various mechanisms, including apoptosis. However, *MTB* can evade host immunity to create a favorable environment for intracellular replication. *MTB*-infected human macrophages produce interleukin-32 (IL-32). IL-32 is a pro-inflammatory cytokine and has several isoforms. We previously found that IL-32γ reduced the burden of *MTB* in human macrophages, in part, through the induction of caspase-3-dependent apoptosis. However, based on our previous studies, we hypothesized that caspase-3-*independent* death pathways may also mediate IL-32 control of *MTB* infection. Herein, we assessed the potential roles of cathepsin-mediated apoptosis, caspase-1-mediated pyroptosis, and apoptosis-inducing factor (AIF) in mediating IL-32γ control of *MTB* infection in THP-1 cells.

**Results:**

Differentiated human THP-1 macrophages were infected with *MTB* H37Rv alone or in the presence of specific inhibitors to caspase-1, cathepsin B/D, or cathepsin L for up to four days, after which TUNEL-positive cells were quantified; in addition, *MTB* was quantified by culture as well as by the percentage of THP-1 cells that were infected with green fluorescent protein (GFP)-labeled *MTB* as determined by microscopy. AIF expression was inhibited using siRNA technology. Inhibition of cathepsin B/D, cathepsin L, or caspase-1 activity significantly abrogated the IL-32γ-mediated reduction in the number of intracellular *MTB* and of the percentage of GFP-*MTB*-infected macrophages. Furthermore, inhibition of caspase-1, cathepsin B/D, or cathepsin L in the absence of exogenous IL-32γ resulted in a trend toward an increased proportion of *MTB*-infected THP-1 cells. Inhibition of AIF activity in the absence of exogenous IL-32γ also increased intracellular burden of *MTB*. However, since IL-32γ did not induce AIF and because the relative increases in *MTB* with inhibition of AIF were similar in the presence or absence of IL-32γ, our results indicate that AIF does not mediate the host-protective effect of IL-32γ against *MTB*.

**Conclusions:**

The anti-*MTB* effects of IL-32γ are mediated through classical caspase-3-dependent apoptosis as well as caspase-3-independent apoptosis.

## Background

Interleukin-32 (IL-32) is a pro-inflammatory cytokine with pleiotropic functions [1]. IL-32 has at least six isoforms (α, β, γ, δ, ε, and ζ) due to alternative mRNA splice variants [2]. IL-32γ is considered the most biologically active isoform with regards to induction of pro-inflammatory cytokines, perhaps because it has no exonic deletions [2]. Previously, we found that apoptosis – which enhances killing of intracellular *Mycobacterium tuberculosis* (*MTB*) in phagocytes [3-8] – was a mechanism by which IL-32γ reduced the intracellular burden of *MTB* in THP-1 macrophages [9]. However, IL-32γ-induced apoptosis and control of *MTB* infection were only partially abrogated by inhibition of caspase-3, indicating that other cell death pathway (s) may also be involved in the anti-*MTB* effects of IL-32γ [9]. Caspase-3-*independent* forms of apoptosis have received increasing recognition, including those triggered by lysosome proteases known as cathepsins and by apoptosis-inducing factor (AIF). Although AIF is a flavoprotein normally found in mitochondria, it mediates apoptosis by a caspase-independent mechanism [10,11]. Importantly, both these alternative apoptotic pathways have been implicated in controlling mycobacterial infections *in vitro* [5,6,12-14], making them candidate pathways for mediating the anti-*MTB* effects of IL-32γ.

A more recently described form of programmed cell death is one that is mediated by inflammasome-associated caspase-1 [15,16]. This form of cell death is inflammatory in nature and is known as pyroptosis (“the falling of fire”) because it is associated with caspase-1 induction of active IL-1β and IL-18 [15]. While pyroptosis has some molecular signatures in common with apoptosis, there are also distinct differences such as requirement of functional caspase-1 with pyroptosis [15,16]. Thus, we investigated whether any of these three caspase-3-independent pathways contribute to the protective effect of IL-32γ against *MTB* in differentiated THP-1 macrophages.

## Methods

### Materials

The human promonocytic cell line THP-1 (TIB-202) was obtained from the American Type Culture Collection (Manassas, VA). PMA was purchased from Sigma-Aldrich (St. Louis, MO). RPMI 1640 cell culture medium was obtained from Cambrex (East Rutherford, NJ). Fetal bovine serum (FBS) was purchased from Atlanta Biologicals (Norcross, GA) and heat-inactivated at 56°C for one hour. THP-1 cells were cultured in RPMI 1640 supplemented with 10% FBS and 2 mM glutamine and were maintained at a concentration of 2–10 ×10^5^ cells/mL. Apoptosis in Situ Detection Kit was purchased from Roche Diagnostic Systems (Indianapolis, IN). Recombinant IL-32γ (confirmed to be LPS-free) and caspase-1 inhibitor (z-WEHD-fmk) were purchased from R&D System, Inc. (Minneapolis, MN). The cathepsin B inhibitor [L-3-*trans-*(propylcarbamoyl) oxirane-2-carbonyl]-L-isoleucyl-L-proline methyl ester (CA-074-Me)], the cathepsin D inhibitor pepstatin A, and the cathepsin L inhibitor [benzyloxycarbonyl-Leu-Leu-Tyr-fluoromethylketone (z-LLY-fmk)] were purchased from Calbiochem-EMD Millipore (Billerica, Massachusetts). The AIF-siRNA kit was purchased from Santa Cruz Biotechnology (Dallas, TX). Polyclonal antibody to AIF and secondary antibody IgG-HRP for Phototope-Western Blot Detection System were purchased from Cell Signaling, Inc (Beverly, MA). ProLong® Gold antifade reagent with DAPI was purchased from Invitrogen (Eugene, OR).

### Mycobacterial culture and reagents

*MTB* H37Rv was obtained from the American Type Culture Collection (27294) and grown to log phase at 37°C in Difco Middlebrook 7H9 Medium (Becton Dickinson, MD) enriched with 10% stock ADC Enrichment (Remel, Lenexa, KS) which contains 5% (w/v) BSA fraction V, 2% (w/v) glucose, 0.87% (w/v) NaCl, and 0.004% (w/v) catalase. In addition, 0.05% (v/v) Tween 80 and 0.2% (v/v) glycerol were added to the growth medium. After culture of the mycobacteria under aeration, the culture was diluted to a concentration of 1.0 McFarland standard (~10^8^ bacilli/mL) and stored at −80°C.

### Infection of macrophages, culture of *MTB*, and microscopy of GFP-*MTB*

Differentiated THP-1 human macrophages were infected with *MTB* H37Rv at a multiplicity-of-infection (MOI) of 10 bacilli to 1 macrophage, and washed after one hour of infection. For the one hour infection time point, the cells were lysed, serial dilution performed, and the lysates were cultured for *MTB* and the colony forming units (CFU) quantified as described [17]. For the two and four day time points, following washing the cells after the one hour infection, fresh medium was added, cultures were incubated for the indicated time points, and cells lysed for quantitation of *MTB*.

To independently validate the CFU data, we also infected THP-1 cells with GFP-labeled *MTB* H37Rv under various experimental conditions and quantified the proportion of THP-1 cells infected with GFP-*MTB* by fluorescent microscopy [17]. Differentiated THP-1 cells (0.4 × 10^5^ cells/well) were infected with GFP-*MTB* H37Rv at an MOI of 10 on four-well chamber slides. After one hour, the cells were washed, cultured for four days at 37°C in 5% ambient CO_2_, and then fixed with 4% paraformaldehyde. The cells were then stained and mounted with ProLong® Gold antifade reagent with DAPI. The images were captured using an inverted Zeiss 200 M microscope (Carl Zeiss, Thornwood, CA). The number of THP-1 cells with internalized GFP-*MTB* was quantified by microscopy, counting at least 300 consecutive macrophages and calculating the percentages of macrophages that contained GFP-*MTB*.

### Tunel assay

Cell suspensions (0.4 × 10^5^ cells in 0.5 mL) were grown on four-chamber well slides, pre-incubated with or without inhibitors to caspase-1 or cathepsins, and then infected with *MTB* H37Rv at an MOI of 10. Scrambled siRNA- or AIF siRNA-transfected THP-1 cells were incubated on chamber slides and then infected with *MTB*. Slides were prepared in triplicate for each condition. The medium was removed at 2 days following infection and the cells were fixed in 4% paraformaldehyde solution (pH 7.4). The cells were then stained with an Apoptosis In Situ Detection Kit according to the manufacturer’s instructions. The number of TUNEL positive cells was reported as percent of total number of cells counted as previously described [17].

### Silencing of AIF and Western blot analysis

THP-1 cells were seeded at a density of 1 × 10^5^ cells per well in 12-well plates, differentiated with 15 ng/mL PMA overnight, and then incubated with fresh medium without PMA. Either 50 pmol of scrambled siRNA or AIF siRNA (in 100 μL siRNA Transfection Medium + 100 μL siRNA Transfection Reagents) was transfected into THP-1 cells according to the manufacturer’s instructions. After transfection, the cells were washed with 1:1 solution of PBS: medium buffer. To confirm inhibition of AIF expression, whole cell lysates were prepared and western blot performed using polyclonal AIF antibody (1:1000 in TBS buffer with 5% BSA) and secondary IgG-HRP (1:2000) as previously reported [9].

### Statistical analysis

Replicate experiments were performed independently, and where appropriate, summary results are presented as means ± SEM. Differences were considered significant for p < 0.05, and all reported p-values used a two-sided test. For most experiments, group means were compared by ANOVA using Fisher’s least significant difference procedure.

## Results

### IL-32-induced pyroptosis reduces intracellular burden of *MTB*

**Figure 1:**
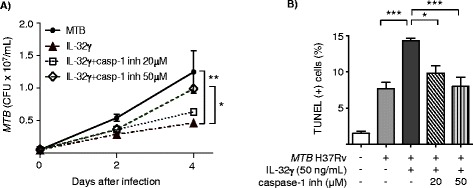
**Caspase-1 contributes to the anti-** ***MTB*** **effects of IL-32γ. A)** THP-1 cells were infected with *MTB* alone, *MTB* + IL-32γ (50 ng/mL), or pre-incubated with a caspase-1 inhibitor for one hour and then incubated with *MTB* + IL-32γ. Intracellular *MTB* were quantified one hour, two days, and four days after infection. **B)** TUNEL positive cells were quantified two days after *MTB* infection ± IL-32γ (50 ng/mL) with and without the caspase-1 inhibitor. Data shown are the mean ± SEM of four independent experiments. *p < 0.05, **p < 0.01, ***p < 0.001. inh = inhibitor.

In order to determine whether caspase-1-dependent pyroptosis contributes to the antagonistic effects of IL-32γ against *MTB* in macrophages, a specific pharmacologic inhibitor to caspase-1 (z-WEHD-fmk) was utilized. THP-1 cells were left untreated or treated with 20 μM or 50 μM z-WEHD-fmk for one hour, then stimulated with 50 ng/mL of recombinant IL-32γ and infected with *MTB* H37Rv. After one hour, two days, and four days of infection, *MTB* was quantified. Compared to control cells, IL-32γ significantly reduced the number of *MTB* recovered at two and four days after infection. With 20 μM caspase-1 inhibitor, there was a partial, insignificant abrogation of IL-32-mediated reduction of *MTB*; however, with 50 μM z-WEHD-fmk, there was a significant abrogation of IL-32γ-mediated reduction of intracellular *MTB* (Figure 1A). Additionally, IL-32-induced apoptosis of *MTB*-infected macrophages was significantly inhibited by the caspase-1 inhibitor (Figure 1B).

### Lysosomal cathepsin-induced apoptosis modestly contributes to IL-32γ antagonism of *MTB*

**Figure 2:**
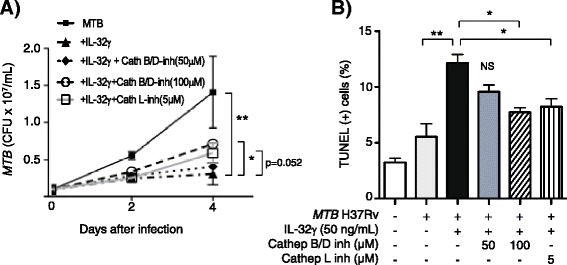
**Cathepsins contribute to the anti-** ***MTB*** **effects of IL-32γ. A)** THP-1 cells were infected with *MTB* alone, *MTB* + IL-32γ (50 ng/mL), or pre-incubated with combined cathepsin B and D inhibitors or with cathepsin L inhibitor for one hour and then incubated with *MTB* + IL-32γ. Intracellular CFU were quantified in one hour, two days, and four days after infection. **B)** TUNEL positive cells were quantified two days after *MTB* infection ± IL-32γ (50 ng/mL) with and without cathepsin B/D inhibitors or cathepsin L inhibitor. Data shown are the mean ± SEM of four independent experiments. *p < 0.05, **p < 0.01.

To determine the contribution of lysosomal cathepsin-induced apoptosis to the inhibitory effect of IL-32γ against *MTB*, we used potent and selective inhibitors to cathepsin B (CA-074Me), cathepsin D (pepstatin A), and cathepsin L (z-LLY-fmk). Since cathepsin B and D are functionally related [18-20], THP-1 cells were pre-incubated with combined CA-074Me and pepstatin A (50 μM or 100 μM of each) for one hour to inhibit cathepsin B and D simultaneously or with 5 μM of z-LLY-fmk to inhibit cathepsin L, and then stimulated with 50 ng/mL IL-32γ and infected with *MTB* H37Rv [21-23]. In the presence of combined cathepsin B and D inhibitors at 100 μM but not at 50 μM, there was significant abrogation of the IL-32γ-induced reduction of intracellular burden at four days after infection (Figure 2A). With inhibition of cathepsin L, there was also abrogation of the IL-32γ-mediated reduction in *MTB* that nearly reached significance (Figure 2A). Inhibition of cathepsin B/D or cathepsin L also significantly reduced the number of TUNEL positive cells induced by IL-32γ and *MTB* (Figure 2B).

### Inhibition of caspase-1, cathepsin B/D, or cathepsin L increased the proportion of *MTB*-infected THP-1 cells

**Figure 3:**
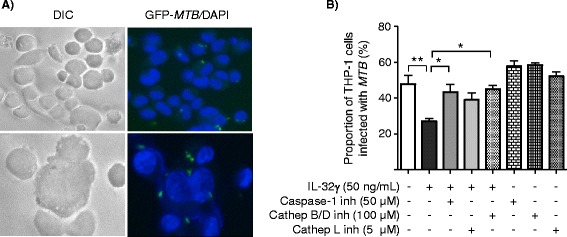
**IL-32 reduces the proportion of** ***MTB*** **-infected THP-1 cells and this effect is partly dependent on caspase-1, cathepsins B/D, and cathepsin L activity.** Differentiated THP-1 cells were infected with GFP-*MTB* H37Rv with the indicated conditions for four days and the proportions of *MTB*-infected cells were quantified by fluorescent microscopy. **A)** Representative DIC and fluorescent microphotograph of control THP-1 cells infected with GFP-labeled *MTB H37Rv* at lower magnification (400×, top) and higher magnification (600×, below). **B)** The proportion of THP-1 cells infected with GFP-*MTB* H37Rv was quantified after four days of culture for the indicated conditions. Data shown are the mean ± SEM of two independent experiments, each performed in duplicate. *p < 0.05, **p < 0.01.

To validate the CFU findings, we infected THP-1 cells with GFP-labeled *MTB* H37Rv in medium alone, 50 ng/mL IL-32γ, or IL-32γ with caspase-1 inhibitor, cathepsin B/D inhibitors, or cathepsin L inhibitor. After four days of incubation, the percentages of GFP-*MTB* infected cells were quantified by microscopy (Figure 3A). As shown in Figure 3B, IL-32γ reduced the percentage of *MTB*-infected macrophages and caspase-1 inhibition (z-WEHD-fmk) significantly abrogated this. Similarly, inhibition of cathepsin B/D (CA-074Me and pepstatin A) or of cathepsin L (z-LLY-fmk) partially abrogated IL-32γ-mediated reduction in *MTB*-infected macrophages. Incubation of GFP-*MTB*-infected macrophages with the inhibitors but without addition of IL-32γ showed a trend toward increase percentage of *MTB*-infected cells (Figure 3B, compare bar 1 with bars 6, 7, and 8).

### AIF is not induced by IL-32γ but its inhibition increases intracellular *MTB*

**Figure 4:**
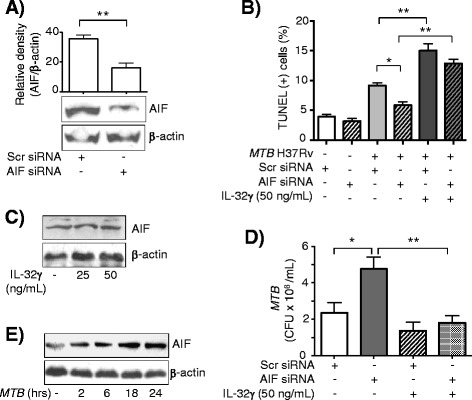
**Apoptosis-inducing factor (AIF) reduces burden of** ***MTB*** **in THP-1 cells but does not mediate the anti-** ***MTB*** **effects of IL-32γ. A)** Western blot analysis for AIF in THP-1 cells transfected with scrambled siRNA and with siRNA that is complementary in sequence to AIF mRNA. Immunoblot and densitometry data shown are representative and the mean ± SD of three independent experiments, respectively. **B)** Apoptosis was quantified by TUNEL in THP-1 cells transfected with scrambled siRNA or AIF siRNA ± IL-32γ (50 ng/mL) ± *MTB* infection for 48 hours. Data shown are the mean ± SEM of three independent experiments. **C)** Immunoblot for AIF in THP-1 cells incubated with IL-32γ for 24 hours. Data shown are representative of three independent experiments. **D)** THP-1 cells transfected with scrambled siRNA or AIF siRNA were incubated with *MTB* ± IL-32γ (50 ng/mL) for four days and *MTB* quantified. Data shown are the mean ± SEM of three independent experiments. **E)** Immunoblot for AIF in THP-1 cells infected with *MTB* at the indicated times. Data shown are representative of three independent experiments. *p < 0.05, **p < 0.01.

In order to study the role of AIF in IL-32γ-stimulated, *MTB*-infected macrophages, siRNA technology was employed to silence AIF expression in THP-1 cells. Western blot analysis confirmed that AIF expression was down-regulated by siRNA-AIF compared to cells transfected with scrambled siRNA (Figure 4A). *MTB* infection of THP-1 cells transfected with the scrambled siRNA increased the number of TUNEL positive cells (Figure 4B, compare bars 1 and 3). However, this induction was significantly abrogated when THP-1 cells were knocked-down for AIF, indicating that *MTB*-induced apoptosis is partly mediated by AIF (Figure 4B, compare bars 3 and 4). In control THP-1 cells exposed to both *MTB* and IL-32γ, there was a further increase in apoptosis but with AIF inhibition, the reduction in apoptosis was similar in the presence or absence of IL-32γ, indicating that AIF does not significantly contribute to IL-32γ-induced apoptosis (Figure 4B, compare relative difference of bars 3 and 4 vs. bars 5 and 6). Indeed, this finding is supported by the fact that AIF is not induced by IL-32γ (Figure 4C).

Since knocking down AIF in THP-1 cells did not significantly abrogate the reduction in CFU by IL-32γ (Figure 4D, compare bars 3 and 4), it further indicated that AIF does not significantly mediate the anti-*MTB* activity of IL-32γ in human macrophages. Reduction of apoptosis with inhibition of AIF in *MTB*-infected cells suggested that *MTB* itself could induce AIF expression. To validate this qualitatively, THP-1 cells were infected with *MTB* for two to 24 hours, whole cell lysates prepared, and immunoblot for AIF performed on the separated proteins. As can be seen in Figure 4E, AIF expression was induced in THP-1 cells after infection with *MTB* for 18 and 24 hours.

## Discussion

Macrophages kill intracellular *MTB* through a variety of mechanisms, including phagosome-lysosome fusion, autophagy, and apoptosis [3,5-7,24-28]. Subsequent ingestion of apoptotic bodies that contain mycobacteria can further enhance antigen presentation to T cells [13]. Phagocytosis of *Mycobacterium avium*-infected apoptotic bodies has also been shown to decrease mycobacterial viability [29]. In addition to caspase-3-mediated apoptosis, other forms of programmed cell death may be utilized by *MTB*-infected macrophages, including apoptosis mediated by caspase-1, cathepsins, and AIF [5,6,12-14].

Previously, we found that IL-32γ reduced the burden of intracellular *MTB* in macrophages through induction of caspase-3-dependent apoptosis. However, since inhibition of caspase-3 only partially abrogated the increased number of TUNEL positive cells induced by IL-32γ, it indicated that caspase-3-independent pathway (s) may also contribute to IL-32γ-induced apoptosis and subsequent control of *MTB*.

IL-32 induces caspase-1 activation and IL-1β production in human macrophages [9]. Since we previously showed that *MTB-*induced caspase-1 activation plays an important role in the production of various inflammatory cytokines including IL-32 [30,31], we used a pharmacological inhibitor of caspase-1 to determine whether this key enzyme of inflammasomes also mediates the anti-*MTB* effects of IL-32γ. We found that inhibiting caspase-1 activity significantly abrogated the IL-32γ-mediated reduction in the number of viable *MTB* as well as reduced the amount of programmed cell death. These findings indicate that caspase-1-mediated pyroptosis is a mechanism that contributes to the anti-*MTB* effect of IL-32γ. Caspase-1 inhibition alone (without addition of exogenous IL-32γ) showed a trend toward an increased proportion of THP-1 macrophages infected with GFP-*MTB*, supporting our finding that caspase-1 activation and pyroptosis contribute to macrophage control of *MTB*. Indeed, there is increasing evidence that pyroptosis plays an important role in host defense against intracellular pathogens such as *Salmonella*, *Shigella*, *Legionella*, *Francisella*, and *Listeria* [15]. Another possible non-mutually exclusive mechanism is that caspase-1 induced IL-1β expression activated macrophages to control *MTB* more effectively [9]. Ciaramella and co-workers also found that infection of human monocytes with *MTB* induced caspase-1-mediated apoptosis as well as TNFα and IL-1β production [32]. The same group of investigators noted that with a high MOI infection (MOI 20), the cell death seen was not associated with reduced *MTB* viability; however, another interpretation is that the high death rate of monocytes at 48 hours is actually protective since 48 hours of unrestricted growth should result in ~ four-fold increase in CFU but instead the total *MTB* burden per well was unchanged compare to the initial Day 0 (three hour) time point [33]. While Master *et al.* [34] showed that *MTB* inhibited the NLRC4 inflammasome, it is clear that other types of inflammasome complexes must be activated due to the production or activation of inflammasome-dependent IL-1β, IL-18, and caspase-1 by macrophages following *MTB* infection [9,30,31,35,36]. Indeed, ESAT-6, a protein secreted by *MTB*, can induce the transcription of caspase-1 [35] and activate the NLRP3 inflammasome [37]. Furthermore, exogenous ATP, a potent activator of the inflammasome and pyroptosis, induces human macrophages to kill intracellular *MTB* [38].

Lysosomes contain many different types of proteases collectively called cathepsins [5]. Many cathepsins are stored as pro-enzymes that only become activated at low pH in phagolysosomes. When released into the cytoplasm, the cathepsin proteases have been shown to mediate both propidium iodide-positive cell death and apoptosis in macrophages following *MTB* infection [5,6,13]. While Lee and colleagues showed that *MTB* Erdman could trigger cell death in murine macrophages that was mediated by cathepsins B and L and that this form of apoptosis did not directly reduce mycobacterial viability, these experiments used a very high MOI of 25 [6]. Nevertheless, when fresh macrophages were added to these *MTB*-infected macrophages early in the apoptotic process, they enhanced killing of *MTB* whereas when fresh macrophages were added to *MTB*-infected macrophages late, when the latter dying cells were undergoing necrosis, increased *MTB* killing was not seen [6]. These findings implicate that *in vivo*, cathepsin-mediated cell death could be host-protective since there would be a steady influx of monocytes and macrophages to the site of the infection. The same investigators also showed that a high inoculum of virulent *MTB* (MOI 25) in murine macrophages induced an atypical form of cell death that displayed ultrastructural features consistent with both apoptosis and necrosis; this form of cell death was independent of caspase-3, caspase-1, and cathepsins but dependent on the PhoPR sensor kinase, and allowed extracellular spread of viable *MTB* [23]. O’Sullivan and colleagues showed in THP-1 cells infected with a less virulent strain of *MTB* (H37Ra) that DNA fragmentation was partially dependent on cathepsin L but not cathepsin B [13]. Furthermore, *MTB* H37Ra infection led to an apoptosis-like cell death that was caspase-independent but mediated by a serine protease [13]. On the other hand, infection with the more virulent *MTB* H37Rv at an MOI of 10–20 induced a form of cell death that was dependent on both caspase and serine proteases [13]. We found that inhibition of cathepsin B/D or cathepsin L modestly abrogated the number of TUNEL positive cells (by ~30 to 40%) induced by IL-32γ and *MTB*, as well as partially reversed the anti-*MTB* effect of IL-32γ. Inhibition of cathepsin B/D or cathepsin L in the absence of exogenous IL-32γ showed a trend toward increased proportion of macrophages infected with GFP-*MTB*, supporting the CFU data that cathepsin activation modestly contributes to macrophage control of *MTB*. These findings indicate that IL-32γ-induced apoptosis is at least partially dependent on the release of lysosomal proteases that augment apoptosis rather than simple facilitation of secondary necrosis. While apoptotic cells eventually undergo necrosis and it is difficult to reliably distinguish this form of secondary necrosis from primary necrosis, we believe that we are measuring mainly apoptosis since the TUNEL assay was performed relatively soon after infection. Collectively, these findings would indicate that a high burden of intracellular *MTB* is more likely to induce a form of cell death that is not host-protective. Our findings would also implicate that IL-32γ is capable of inducing cell death phenotypes (classical apoptosis, pyroptosis, and cathepsin-mediated cell death) that enhanced macrophage control of moderate burden of *MTB*. Compared to studies that infected with a MOI of 20 to 25 – which results in an intracellular bacillary load of ~20 bacilli per macrophage – it is important to note that the MOI of 10 we employed is likely to result in an intracellular burden that is comparatively even lower because we washed the cells after one hour of infection whereas the high MOI studies infected the cells for three hours before washing [23,33].

AIF is a flavoprotein normally found in the inter-membranous space of mitochondria. In response to specific death stimuli, AIF is released into the cytoplasm, and translocates into the nucleus to recruit endonucleases that cause DNA fragmentation and chromatin condensation [10,11]. Vega-Mariquez and colleagues showed that bovine macrophages infected with *Mycobacterium bovis* underwent programmed cell death via a caspase-independent, AIF-mediated mechanism [14]. Evidence from our studies would indicate that AIF does not mediate the protective effects of IL-32γ against *MTB*. First, similar reduction in apoptosis in cells knocked-down for AIF with either exposure to *MTB* alone or to *MTB* plus IL-32 suggests IL-32-induction of apoptosis is not through AIF. Second, inhibiting AIF expression did not significantly abrogate either IL-32 increase in apoptosis or IL-32-induced reduction in CFU. Third, IL-32γ did not induce AIF expression.

## Conclusions

**Figure 5:**
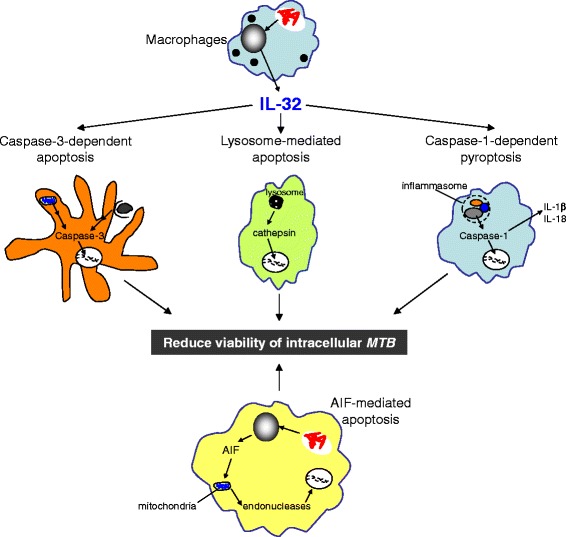
**Cell death pathways hypothesized to be involved in IL-32 control of** ***MTB*** **infection in macrophages.** Macrophages can undergo apoptosis through various mechanisms including those mediated by caspase-3, caspase-1, cathepsins, and AIF. While all four pathways contribute to macrophage control of *MTB* infection, caspase-3, caspase-1, and cathepsins, but not AIF, mediate the anti-*MTB* effects of IL-32γ in macrophages. Fragmented DNA is used to depict the various forms of programmed cell death. Both extrinsic and intrinsic pathways of classical apoptosis are shown in the caspase-3-dependent apoptosis. *MTB* are illustrated by red bacilli in phagosomes.

In conclusion, we determined that in addition to caspase-3-dependent apoptosis, IL-32γ also induces apoptosis of *MTB*-infected macrophages through other mechanisms including those mediated by caspase-1 and lysosomal cathepsins (Figure 5). Although AIF-mediated apoptosis was also identified to be an important anti-*MTB* mechanism, AIF does not appear to mediate the anti-*MTB* effects of IL-32γ. Our findings contribute to the increasing recognition that apoptosis of *MTB*-infected macrophages plays an important host-defense role against *MTB*.
